# Integrating mental health screening into routine community maternal and child health activity: experience from Prevention of Mother-to-child HIV transmission (PMTCT) trial in Nigeria

**DOI:** 10.1007/s00127-014-0952-7

**Published:** 2014-09-09

**Authors:** Theddeus Iheanacho, Michael Obiefune, Chinenye O. Ezeanolue, Gbenga Ogedegbe, Okey C. Nwanyanwu, John E. Ehiri, Jude Ohaeri, Echezona E. Ezeanolue

**Affiliations:** 1 Department of Psychiatry, Yale Univeristy School of Medicine, 300 George Street, New Haven, CT 06519 USA; 2 Prevention, Education, Treatment, Training and Research-Global Solutions-PeTR-GS, 7 Link Road Independence Layout Enugu, 400001 Enugu State Enugu, Nigeria; 3 Sunrise Foundation, Plot 358 New GRA, Enugu, Nigeria; 4 School of Medicine, New York University, 550 1st Ave, New York, NY USA; 5 United States Center for Diseases Control and Prevention, Country Office, Abuja, Nigeria; 6 Division of Health Promotion Sciences/Global Health Institute, Mel and Enid Zuckerman College of Public Health, University of Arizona, 1295 N. Martin Ave., Tucson, AZ 85724 USA; 7 Department of Psychological Medicine, University of Nigeria Teaching Hospital, Enugu, Nigeria; 8 Department of Pediatrics, University of Nevada School of Medicine, 2040 West Charleston Boulevard, Las Vegas, NV USA

**Keywords:** Mental health screening, General Health Questionnaire 12, Community and church-based screening, Adult Nigerians, Low- and middle-income countries (LMIC), PMTCT, Maternal and child health

## Abstract

**Purpose:**

Although the prevalence of mental health disorders in Nigeria is comparable to most developed countries, access to mental health care in Nigeria is limited. Improving access to care requires innovative approaches that deliver mental health interventions at the community level. The aim of this study was to determine the feasibility and acceptability of integrating mental health screening into an existing community-based program for prevention of mother-to-child transmission of HIV targeted at pregnant women and their male partners.

**Methods:**

Pregnant women and their male partners from 117 churches enrolled in the healthy beginning initiative (HBI) in southeast Nigeria participated in the mental health screening project. Two members from each church were trained as church-based health advisors to administer the 12-item general health questionnaire.

**Results:**

Ninety-three percent of the pregnant women and their male partners agreed to participate and fully completed the questionnaire. Overall, 21.7 % of the respondents scored above the threshold of 11 indicating significant psychological distress, with women having significantly higher scores than men.

**Conclusion:**

Mental health screening is feasible and well accepted among a cohort of pregnant women and their male partners. Church members can be trained as health advisors to administer mental health screening. Mental health interventions can be developed on the framework of the HBI.

## Introduction

### Burden of mental health in Nigeria

Nigeria exemplifies the severe gap in mental health care common in low- and middle-income countries (LMIC) [1] with an estimated 150 psychiatrists available in a country of 160 million people. Published studies show that up to 26 % of adult Nigerians had a lifetime prevalence of at least one DSM IV disorder [2–4]. An estimated 10 % of Nigerian women experience depression during pregnancy while approximately 20 % of women experience post-natal depression [5, 6]. Poor maternal mental has been shown to negatively impact a child’s physiological, emotional and cognitive development [6, 7] and is a major contributor to maternal mortality [8–10].

### Access to mental health in Nigeria

There are limited data from Nigeria on maternal access to mental health care during and after pregnancy. However, available studies indicate that only about 10 % of adults with mental illness in Nigeria receive any care irrespective of severity [2]. Mental health care during pregnancy is scarce and is limited to major urban hospitals. However, more than 50 % of pregnant women in urban areas and more so in rural parts of Nigeria seek perinatal services in non-hospital settings; usually from untrained and unregulated traditional birth attendants [11, 12]. Conversely, most pregnant women and their families get support from their religious organizations during pregnancy [11, 13]. Religious organizations offer special prayer sessions for pregnant couples and celebrate with the families at special services after childbirth [14].

### The healthy beginning initiative

The Healthy Beginning Initiative (HBI), funded by the United States National Institute of Health (NIH), was designed to assess the effectiveness of a church-based program that uses prayer sessions to recruit pregnant women early in their pregnancy, utilizes baby shower activities to deliver multiple health interventions (including health education and integrated laboratory testing), and baby receptions to follow up with the women and link them to continuing care after delivery [15]. The rational to use congregation-based approach was based on several reasons: first, Nigeria has extensive penetration of faith-based institutions and faith plays a significant role in the social life of Nigerians. Second, Nigeria is ranked #1 among 53 other nations in church attendance with 89 % of sampled Christian population reporting at least once a week attendance [16–18]. Third, the use of a family-centered, culturally appropriate approach that relies on a widely distributed infrastructure (religious institutions), if effective, could easily become an attractive model for other maternal and child health interventions in settings that provide interventions to prevent mother-to-child transmission of HIV under the Presidents Emergency Plan for AIDS Relief (PEPFAR) [22]. Finally, the collaborative, community-based approach could strengthen trust between faith-based organizations (FBOs) and researchers at treatment centers, which could make it easier to track enrolled pregnant women and reduce loss to follow-up.

Recent studies estimated that 69 % of adults surveyed in southern Nigeria sought help from their churches, mosques or traditional healers before going to see a healthcare provider [19, 20]. Finding innovative ways to utilize these widely available institutions to enhance access to mental health care that is culturally appropriate is essential to reach the WHO Millennium Development Goals [21, 22]. There is little evidence, however, on the feasibility, acceptability or effectiveness of integrating mental health screening, assessment or treatment into routine religious activities in Nigeria.

### Purpose of study

This pilot study was designed to test the feasibility of integrating mental health screening into a routine community health program. The primary aim was to assess the acceptability of mental health screening among pregnant women and their male partners during the HBI to prevent mother-to-child transmission of HIV. The secondary aim was to assess the feasibility of using trained church-based health advisors to administer a mental health screening instrument. The study was approved by the Institutional Review Board of the University of Nevada, Reno and the Nigerian National Health Research Ethics Committee.

## Methods

### Study setting

Self-identified pregnant women and their partners who attended churches participating in the HBI were approached to participate.

### Study procedure

A detailed study procedure has been described elsewhere [23]. In summary, we recruited and trained 144 mostly female church-based health advisors (CHAs) with an average age of 30 years and a minimum of high school education to administer an investigator-assisted questionnaire to HBI participants. CHAs first received specific training on completing the 12-item General Health Questionnaire (GHQ-12) and translating the questions to the local Ibo language so they can provide assistance and respond appropriately to participants’ inquiries while completing the questionnaire. In addition to providing assistance to the participants in completing the GHQ-12, the CHAs provided education to the families on medical screening, breastfeeding, mother–child bonding, mental health and nutrition.

All the bishops who oversaw churches in the proposed study areas agreed to participate in this study following detailed discussions and explanations from the research team. Recruitment occurred at the level of the churches and participants (in that order). Participant’s recruitment followed four steps: (1) potential participants were approached and provided with detailed explanation of the study and its objectives. (2) Those who expressed interest were asked to read the written consent form, allowed to ask questions and were assured of confidentiality. (3) Participants were then asked to sign the written consent form as a confirmation of their voluntary participation in the study. (4) For participants who are unable to read and write, the consent was read to them in the native language, by the CHA and research assistant. After all their questions about the study had been addressed, those who volunteered to participate were asked to give their thumb print as a confirmation of their informed consent.

Participants were clearly informed that HBI is not a substitute for prenatal care and were encouraged to follow up with their healthcare provider for regular care during pregnancy.

### Questionnaire administration and completion

The English version of the GHQ-12 questionnaire was used for this study. We decided against translation to the local language (Ibo) after it was noted that although participants were able to speak the local language, majority of the participants were unable to read the local language and preferred to complete the questionnaire in English. Trained research assistants and church-based health advisors selected from participating churches assisted participants with completion of the questionnaire. For participants who could not read, the trained church-based health advisor will read out the questions to them. For participants who do not understand Standard English, the church-based health advisor will first read out the question in English and translate to the local language.

### Data analysis

#### Measures

The GHQ-12 is a validated and reliable 12-question screening instrument that detects general psychological distress and has been used in multiple studies in Nigeria [24]. Each item on the scale has four responses from “better than usual” to “much less than usual”. There are several ways of scoring this measure none being particularly better than the others [25]. For the purpose of this study, we choose the Likert scoring (0–1–2–3) method which gives a possible score range of 0–36. Likert scoring produces superior score distribution to assess severity when psychiatric disorders are considered as dimensions rather than categories [26]. Due to population variations in thresholds for the GHQ-12, the mean GHQ score for a population of respondents is suggested as a rough estimator of the best cut-off point [27]. Higher scores on the GHQ-12 indicate greater levels of general psychological distress [28]. The mean GHQ-12 score for this sample was 8.71 (SD = 5.0) and the threshold score for a high probability of significant psychological distress was 11 (75th Percentile) [26, 27]. This threshold was chosen based on previous studies using GHQ-12 among adult populations in Nigeria [29, 30].

### Statistical analysis

Descriptive statistics was used to examine the demographic characteristics of the sample as well as their GHQ-12 scores. Chi square and independent *T* tests were used to determine if there were any significant gender differences. Fisher’s exact test was used when Chi square tests were inappropriate because of small expected frequencies. *P* values less than .05 were considered significant. All analysis was performed using SAS software version 9.1 (SAS Institute Inc., NC).

## Results

In all, 93 % (*n* = 4,747) of the pregnant women and their male partners who consented to participate in this study completed the GHQ-12 questionnaire. Most of those who did not complete the questionnaire were the male partners who anecdotally reported to field staff that they felt that this survey was more “for the women”. To assess whether completers differed significantly from non-completers, we followed up a sample of 20 non-completers. Our review of their socio-demographic characteristics did not suggest that non-completers were markedly different from completers. A majority of the participants were married (96.4 %), had post-primary education (66.8 %) and some form of employment (52.7 %). A larger proportion were female *n* = 2,796 (55.2 %) with the mean age of 28.68 (SD = 6.5). Women participants were younger (28.68 vs. 37.07 years old), less likely to be married (93.5.9 vs. 99.9 %), more likely to be unemployed (58.3 vs. 33.7 %), more likely to have secondary education (56.7 vs. 44.3 %) and more likely to be living in rural areas (71.3 vs. 67.9 %). There were no significant gender differences in the number of people in the household, distance to closest health facility, awareness of sickle cell trait status or HIV status. The mean GHQ-12 score for the sample was 8.71 (SD = 5.0) (Table 1).

**Table 1:** Sample demographics and characteristics

Variable	Female *n* (%)	Male *n* (%)	Total *n* (%)	Significance
Marital status				
Married	2,615 (93.5 %)	2,305 (99.9 %)	4,920 (96.4 %)	*χ* ^2^ (2) = 149.13, *p* <0.001
Single	179 (6.4 %)	2 (0.1 %)	181 (3.5 %)	
Divorced	2 (0.1 %)	0 (0.0 %)	2 (0.0 %)	
Education				
None	50 (1.8 %)	70 (3.1 %)	120 (2.3 %)	*χ* ^2^ (3) = 146.49, *p* <0.001
Primary	688 (24.2 %)	891 (38.9 %)	1,579 (30.8 %)	
Secondary	1,608 (56.7 %)	1,013 (44.3 %)	2,621 (51.1 %)	
Tertiary	492 (17.3 %)	314 (13.7 %)	806 (15.7 %)	
Employment				
Full time	769 (28.0 %)	1,131 (50.7 %)	1,900 (38.2 %)	*χ* ^2^ (2) = 326.65, *p* <0.001
Part time	374 (13.6 %)	348 (15.6 %)	722 (14.5 %)	
Unemployed	1,599 (58.3 %)	751 (33.7 %)	2,350 (47.3 %)	
Area of residence				
Urban	813 (28.7 %)	737 (32.1 %)	1,550 (30.2 %)	*χ* ^2^ (1) = 7.04, *p* <0.01
Rural	2,023 (71.3 %)	1,560 (67.9 %)	3,583 (69.8 %)	
Closest health facility				
0–5 km	943 (33.9 %)	838 (37.6 %)	1,781 (35.6 %)	*χ* ^2^ (3) = 146.49, *p* <0.001
5–10 km	1,072 (38.5 %)	829 (37.2 %)	1,901 (38.0 %)	
10–15 km	477 (17.1 %)	333 (15.0 %)	810 (16.2 %)	
15+ km	290 (10.4 %)	226 (10.2 %)	516 (10.3 %)	
Age	Female	Male	Total	Significance
Mean	28.68	37.07	32.68	F (1, 4,628) = 302.50,
SD	6.5	22.7	16.9	*p* < 0.001, *η* ^2^ =0.061
N	2,424	2,206	4,630	
GHQ score				
Mean	9.10	8.23	8.71	F (1, 4,745) = 36.07,
SD	5.1	4.8	5.0	*p* <0 .001, *η* ^2^ = 0.008
N	2,620	2,127	4,747	

Overall, 21.7 % of the respondents scored above the threshold of 11 indicating significant psychological distress (Table 2). About 3 % scored 20 or over, indicating severe psychological distress. The level of psychological distress in the pregnant women was higher than in their male partners as shown by a significantly higher mean score (9.10 vs. 8.23 *p* < 0.0001) (Table 1). These findings seemed to indicate that the women were slightly more vulnerable to develop and experience psychiatric disorders perhaps related to pregnancy. In general, higher scores on the GHQ-12 were significantly correlated with being single (*χ* ^2^ (2) = 23.67, *p* < 0.001), having only primary education (*χ* ^2^ (3) = 16.29, *p* < 0.01), being unemployed (*χ* ^2^ (2) = 15.04, *p* < 0.01) and not knowing their sickle cell trait status (*χ* ^2^ (1) = 8.42, *p* < 0.01). There were no significant associations between high scores on the GHQ-12 and HIV status, sickle cell trait status, age or number of children in the household (Table 3).

**Table 2:** Frequency of distribution of GHQ-12 scores for the total sample

	*n*	%
Less than 4	449	8.6
4	342	6.5
5	422	8.1
6	785	15.0
7	381	7.3
8	368	7.0
9	338	6.4
10	272	5.2
11	251	4.8
12	217	4.1
13	157	3.0
14	147	2.8
15	119	2.3
16	97	1.9
17	86	1.6
18	71	1.4
19	55	1.0
20	46	0.9
21 and greater	144	2.7
Missing	495	9.4
Total	5,242	100.0

**Table 3:** Correlates of high and low GHQ-12 scores

Variable	GHQ-12 Score below 11		GHQ-12 Score above 11		Significance
	*N*	%	*N*	%	
HIV status					
Negative	996	64.4	550	35.6	*χ* ^2^ (1) = 0.333
Positive	26	72.2	10	27.8	*p* > 0.05
Aware SC trait status					
No	994	67.8	471	32.2	*χ* ^2^ (1) = 8.42
Yes	2,363	72.0	919	28.0	*p* <0.01
SC trait					
AA normal	1,993	72.6	754	27.4	*χ* ^2^ (3) = 3.04
AS carrier	369	69.1	165	30.9	
SS disease	3	75.0	1	25.0	*p* > 0.05
Other	5	62.5	3	37.5	
Marital status					
Married	3,197	71.3	1,289	28.7	*χ* ^2^ (2) = 23.67
Single	93	55.7	74	44.3	*p* < 0.001
Divorced	0	0.0	2	100.0	
Education					
None	77	72.6	29	27.4	*χ* ^2^ (3) = 16.29
Primary	995	68.3	462	31.7	*p* <0.01
Secondary	1,677	70.3	708	29.7	
Tertiary	568	76.4	175	23.6	
Employment					
Full time	1,296	73.8	461	26.2	*χ* ^2^ (2) = 15.04
Part time	466	70.2	198	29.8	*p* < 0.01
Unemployed	1,457	68.1	683	31.9	
# of children					
Mean	2.93		3.53		F (1,105) = 2.54
SD	1.8		1.7		*p* >0.05
N	71		34		
Age					
Mean	32.90		32.16		F (1, 4,226) = 1.53
SD	20.0		8.5		*p* > 0.05
N	3,026		1,202		

A proportion of respondents either did not answer all the 12 items or provided more than one choice for each question. These were grouped under missing data (9.4 %, Table 2).

## Discussion

This pilot study focused primarily on testing the feasibility and acceptability of conducting mental health screening during routine church-based programs for pregnant women in Enugu, southeastern Nigeria. Having a mental health problem in Nigeria remains a taboo and its acknowledgment is fraught with stigmatization. This limits health seeking from psychiatric professionals [31–34]. The result is that most Nigerians with emotional distress and common mental health disorders seek help and support from their churches, spiritual healers and traditional medicine men [19, 35]. Introducing mental health screening as part of a holistic approach to wellness through this faith-based approach goes a long way to both raising awareness and de-stigmatizing mental illness and can make mental health interventions more accessible and acceptable. There were very few women who declined to complete the screening questionnaire. We interviewed a random sample of participants and although a detailed qualitative analysis of respondents is being done at this time, initial analysis and anecdotal reports show a reasonably high uptake of the program and surveys. One important aspect of this program was the buy-in by the church leaders who viewed it as a significant support for their parishioners at a happy but vulnerable time. They also believed that it was a capacity building process for their church-based lay workers. In addition, integrating the screening with other laboratory tests done in the church was normalizing and reduced the stigma otherwise attached to seeing a psychiatric specialist or going to a psychiatric clinic. The rate of psychological distress reflected in these findings is in keeping with the rate in previous surveys and studies which were primarily conducted in medical settings [36–38].

Some limitations of this pilot study are worth pointing out. The initial protocol did not include a second screener or assessment after this initial GHQ-12 test for those who scored above the 25th percentile to determine the true prevalence of psychological distress or psychiatric disorder. At the beginning of the study, we concluded with the church leadership that participants will be referred to the nearest medical facility for pregnancy care. Participants who were identified with high GHQ-12 scores were referred for evaluation with their regular provider or to the closest psychiatric facility at the University of Nigeria Teaching Hospital. However, during the study, we noted an acute shortage of psychiatric clinicians and have initiated a process with the psychiatric department at the University of Nigeria to train priests as first-line counselors with a referral mechanism with the University teaching hospital.

Another limitation is the absence of a formal evaluation of the CHA’s competence in administering the survey and respondents’ rating of their experiences in this innovative program. However, as shown by the very high level of response from participants and anecdotal field reports, participants and staff alike had a generally positive view of the process. Moreover, the most important question for this pilot study was whether these mental health screenings can be done during routine church-based activities and whether this cohort of women and their partners would be willing to complete a mental health screening questionnaire during these routine church activities. The authors are currently completing qualitative analysis of respondents’ satisfaction surveys and exit interviews and plan to add evaluation tools including pre and post tests, for CHAs and clergy training in subsequent proposals.

## Conclusion

Findings from this pilot study indicate that mental health screening can be integrated into a community-based program designed to improve health outcomes for pregnant women. It also showed that lay church-based health advisors with no previous psychiatric training can be trained to deliver mental health screening survey instruments in church settings. Community-based approaches such as the HBI can become platforms for developing interventions to integrate mental health assessment into community program and linkage to care at primary-care centers.
